# Development and Methodological Validation of a Modified Staging System for de Novo Metastatic Breast Cancer

**DOI:** 10.1001/jamanetworkopen.2024.2174

**Published:** 2024-03-13

**Authors:** Tobias Berg, Maj-Britt Jensen, Maria Rossing, Troels Bechmann, Frede Donskov, Ann Søegaard Knoop, Bent Ejlertsen

**Affiliations:** 1 Danish Breast Cancer Group, Department of Oncology, Copenhagen University Hospital, Rigshospitalet, Copenhagen, Denmark; 2 Department of Oncology, Copenhagen University Hospital, Rigshospitalet, Copenhagen, Denmark; 3 Center for Genomic Medicine, Copenhagen University Hospital, Rigshospitalet, Copenhagen, Denmark; 4 Department of Clinical Medicine, Faculty of Health and Medical Sciences, University of Copenhagen, Copenhagen, Denmark; 5 Department of Oncology, Regional Hospital West Jutland, Herning, Denmark; 6 Department of Oncology, Southern Denmark University Hospital, Esbjerg, Denmark; 7 Department of Regional Health Research, University of Southern Denmark, Odense, Denmark

## Abstract

**Importance:**

Validation of a new method for prognostication of de novo metastatic breast cancer (dnMBC) to better reflect the heterogenecity of the disease.

**Objective:**

To perform external methodological validation of the Plichta staging system, a novel prognostic system for de novo metastatic breast cancer (dnMBC).

**Design, Setting, and Participants:**

This retrospective cohort study used a multicenter, nationwide, population-based Danish Breast Cancer Group database to validate the new method. Participants were patients with dnMBC diagnosed between 2010 and 2019. Data were analyzed from April to June 2023.

**Main outcomes and measures:**

A recursive partitioning analysis (RPA) was performed, as demonstrated by Plichta and colleagues, to group patients with similar overall survival (OS) based on clinical factors. The main outcome was to group patients into 4 prognostic groups based on 3-year OS as stage IVa, greater than 70%; stage IVb, 50% to 70%; stage IVc, 25% to less than 50%; or stage IVd, less than 25%. Bootstrapping was applied for 1000 iterations, with final stage assignments based on the most commonly occurring assignment.

**Results:**

A total of 1859 women were included with a median (IQR) age of 69 (57-77) years. With a median potential follow-up of 89.9 (95% CI, 86.4-95.1) months and a median OS of 31.7 (95% CI, 29.5-34.1) months, the RPA stratified patients into 10 groups, with organ sites, estrogen receptor status, and human epidermal growth factor receptor 2 status as the key clinical factors. Three-year survival rates ranged from 62% (95% CI, 56%-69%) to 8% (95% CI, 3%-21%), which were further combined into 3 stage groups: IVb, 59.4% (95% CI, 56.2%-62.8%); IVc, 39.4% (95% CI, 36.2%-43.0%); and IVd, 15.4% (95% CI, 11.2%-21.3%) (*P* < .001). Following bootstrapping, an IVa group emerged, resulting in 4 stage groups with separate 3-year OS rates identified as IVa, 75.8% (95% CI, 67.8%-84.7%); IVb, 58.8% (95% CI, 55.5%-62.3%); IVc, 39.2% (95% CI, 35.8%-43.0%); and IVd, 14.4% (95% CI, 10.8%-19.4%) (*P* < .001).

**Conclusions and relevance:**

These findings provide external and independent validation of the methods applied in the novel Plichta staging system for dnMBC. This could guide future revisions of the current American Joint Committee on Cancer staging guidelines and may be incorporated as a stratification factor in clinical trials.

## Key Points

**Question:**

Is a new method for prognostication of de novo metastatic breast cancer valid in an external cohort?

**Findings:**

In this cohort study of 1859 participants, the new method, when applied to a Danish, nationwide cohort of patients with de novo metastatic breast cancer, divided patients with stage IV cancer into prognostically distinct subgroups.

**Meaning:**

These findings provide additional robustness to the implementation of the new system by the American Joint Committee on Cancer staging system for breast cancer.

## Teaser

This cohort study performs an external validation of a novel system for understanding outcomes of de novo metastatic breast cancer.

## Introduction

Patients with metastatic disease at the time of initial presentation (de novo metastatic breast cancer [dnMBC]) generally have a poor prognosis. However, most prognostic tools and most clinical trials including patients with metastatic disease do not discriminate between patients with dnMBC and patients that develop subsequent metachronous metastases. This may mask the true outcomes of patients with dnMBC.^1^

Breast cancer staging has, for decades, been solely based on anatomical information until the 2016 revision of the American Joint Committee on Cancer (AJCC), which now includes histological grade, estrogen receptor (ER), progesterone receptor (PR), and human epidermal growth factor receptor 2 (*ERBB2*).^2^ However, no differentiations were added for patients who were diagnosed with de novo metastatic breast cancer, and these patients remain stage IV regardless of histopathological factors.

A recently published staging system has provided detailed insight into the outcomes of patients with dnMBC, in which a total of 42 476 patients with dnMBC from the National Cancer Database were analyzed.^3^ Plichta and colleagues used recursive partitioning analysis (RPA) to identify 53 characteristic profiles based on clinical factors. These characteristic profiles were then evaluated for 3-year OS and afterwards grouped in stage IVa, greater than 70%; IVb, 50% to 70%; IVc, 25% to less than 50%; or IVd, less than 25%. Survival among the individual characteristic profiles ranged from 73.5% to 5.7%, thus reflecting the true heterogeneity in dnMBC.^3^

The methods used in the Plichta-based system warrant external validation to confirm their usefulness and applicability in a modified dataset. Therefore, we conducted an independent validation of Plichta and colleagues’ methods in a clinical, nationwide population of patients with dnMBC.

## Methods

### Data Sources

The Danish Breast Cancer Group’s (DBCG) clinical database contains information on more than 95% of all patients with breast cancer diagnosed in Denmark after 1997. Data on demographics, diagnostics, treatment, and follow-up are registered via electronic report forms and automatic data capture. The DBCG database is updated with information on vital status from the Civil Registration System to ensure complete follow-up.^4^ Eligible criteria for the current study were women aged 18 years or older, with a first invasive breast cancer diagnosed between 2010 and 2019, and metastatic disease at diagnosis or within 90 days. Institutional review board approval was given by the Capital Region’s Center for Health, and consent was waived as most patients were deceased. This study was presented following the Strengthening the Reporting of Observational Studies in Epidemiology (STROBE) reporting guidelines for observational studies.

### Model Development

We used the methods as described by Plichta and colleagues.^3^ Overall survival (OS) was defined as time from diagnosis of M1 disease (dnMBC) until death from any cause. Patients alive on May 1, 2023, were censored at that date. RPA was used with the following clinical variables: T stage (T4 or T0-T3), grade (1-2 or 3), ER status (positive or negative), *ERBB2* status (positive or negative), histologic findings (ductal, lobular, or other), bone-only disease (yes or no), brain-only disease (yes or no), visceral metastases (yes or no), and number of organ systems with metastatic disease (S1, S2, or S3-S4, following Plichta’s end-model). S1 to S4 was defined as the number of organ systems with metastases, so a patient with bone-only disease was assigned S1 and a patient with liver and lung metastases was assigned S2 regardless of the number of liver and lung metastases. The DBCG database contains more granular information on metastatic sites than the National Cancer Database used in Plichta’s system but was simplified to cover the same categories (lung, liver, brain, bone, and other). However, progesterone status (PR) was not available in the DBCG database as PR scoring has not been mandatory since 2010. ER and *ERBB2* status were as defined by American Society of Clinical Oncology and College of American Pathologists guidelines.^5,6^ RPA is a form of multivariate analysis that creates a hierarchical tree of prognostic factors based on predetermined splitting criteria. The decision tree is built based on the most important factor, the second most important factor, and so forth, until reaching a predetermined stopping point. Nodes in RPA refer to groups at a given split. The splitting criteria of the recursive partitioning analysis was set to *P* = .10 with a minimum node size of 50.^7^ After recursive partitioning, 3-year OS rates were estimated for each terminal node, and patients were grouped based on their OS rate according to Plichta and colleagues; group IVa, less than 70%; group IVb, 50% to 70%; group IVc, 25% to less than 50%; and group IVd, less than 25%.

Bootstrapping was subsequently applied with 1000 iterations. For each iteration, a random sample was drawn with replacement from the original data, the RPA was repeated, and each patient characteristic profile was regrouped in IVa to IVd. The final stage assignment was based on the most frequently assigned group by bootstrapping.

### Statistical Analysis

Patient demographics and disease variables were described with numbers and percentages for categorical variables and medians for age. Overall survival was calculated by Kaplan-Meier method, and groups were compared by the log-rank test. Alive patients were censored on May 1, 2023. The potential median follow-up was calculated by Schemper and Smiths’ method of reverse Kaplan-Meier.^8^ Median OS and follow-up are presented with 95% CI. No patients were lost to follow-up. All testing was 2-sided, and a *P* value of .05 was considered significant.

Since the time encompasses several changes in treatment options, especially for *ERBB2*-positive and luminal dnMBC, we calculated OS for the entire cohort and for patients diagnosed between 2010 and 2014 and 2015 and 2019, respectively. All statistical analyses were done using R version 4.3.0 (R Project for Statistical Computing), and the partykit package was used for both RPA and bootstrapping. Data were analyzed from April to June 2023.

## Results

Between January 2010 and December 2019, 1923 women were diagnosed with dnMBC in Denmark and registered in the DBCG database. Analysis of *ERBB2* status was not performed in 64 patients (3%), resulting in a final cohort size of 1859 patients, of whom 1501 have died at follow-up (eFigure 1 in Supplement 1). The median potential follow-up was 89.9 (95% CI, 86.4-95.1) months, and median OS was 31.7 (95% CI, 29.5-34.1) months. The median (IQR) age was 69 (57-77) years.

A total of 475 patients (25.6%) were diagnosed with T4 tumors, and half had visceral metastases at diagnosis. A total of 226 patients (12.1%) had ER- and *ERBB2*-negative tumors, 424 (22.8%) were *ERBB2*-positive, and the remainder were ER-positive, *ERBB2*-negative (1209 patients [65.1%]). Only 12 patients (0.6%) were diagnosed with brain-only disease (Table 1). The RPA stratified patients into 10 groups with a 3-year OS point estimate from 62% (95% CI, 56%-69%) to 8% (95% CI, 3%-21%) (Table 2; eFigure 2 in Supplement 1).

**Table 1.** Baseline Characteristics for all Patients With de Novo Metastatic Breast Cancer 2010 to 2019

Baseline characteristic	Patients, No. (%)				
	All (N = 1859)	Stage IVa (n = 99)	Stage IVb (n = 801)	Stage IVc (n = 696)	Stage IVd (n = 263)
Age, median (IQR), y	69 (57-77)	68 (57-76)	69 (57-77)	70 (59-77)	69 (55-77)
Clinical T-stage					
T0	87 (4.7)	4 (4.0)	42 (5.2)	29 (4.2)	12 (4.6)
T1	288 (15.5)	26 (26.3)	155 (19.4)	79 (11.4)	28 (10.6)
T2	679 (36.5)	47 (47.5)	368 (45.9)	178 (25.6)	86 (32.7)
T3	330 (17.8)	22 (22.2)	152 (19.0)	102 (14.7)	54 (20.5)
T4	475 (25.6)	0	84 (10.5)	308 (44.3)	83 (31.6)
Histologic Finding					
Ductal	1463 (78.7)	81 (81.8)	575 (71.8)	577 (82.9)	230 (87.5)
Lobular	253 (13.6)	0	152 (19.0)	92 (13.2)	9 (3.4)
Other	143 (7.7)	18 (18.2)	74 (9.2)	27 (3.9)	24 (9.1)
Grade					
1	448 (24.1)	20 (20.2)	239 (29.8)	149 (21.4)	40 (15.2)
2	957 (51.5)	67 (67.7)	384 (47.9)	385 (55.3)	121 (46.0)
3	454 (24.4)	12 (12.1)	178 (22.2)	162 (23.3)	102 (38.8)
ER					
Negative	416 (22.4)	46 (46.5)	80 (10.0)	65 (9.3)	225 (85.6)
Positive	1443 (77.6)	53 (53.5)	721 (90.0)	631 (90.7)	38 (14.4)
*ERBB2*					
Negative	1435 (77.2)	18 (18.2)	631 (78.8)	574 (82.5)	212 (80.6)
Positive	424 (22.8)	81 (81.8)	170 (21.2)	122 (17.5)	51 (19.4)
IHC					
ER-negative/*ERBB2*-negative	226 (12.2)	0	0	26 (3.7)	200 (76.0)
ER-positive/*ERBB2*-negative	1209 (65.0)	18 (18.2)	631 (78.8)	548 (78.7)	12 (4.6)
*ERBB2*-positive	424 (22.8)	81 (81.8)	170 (21.2)	122 (17.5)	51 (19.4)
Bone					
No	553 (29.7)	48 (48.5)	255 (31.8)	137 (19.7)	113 (43.0)
Yes	1306 (70.3)	51 (51.5)	546 (68.2)	559 (80.3)	150 (57.0)
Lung					
No	1220 (65.6)	81 (81.8)	670 (83.6)	353 (50.7)	116 (44.1)
Yes	639 (34.4)	18 (18.2)	131 (16.4)	343 (49.3)	147 (55.9)
Liver					
No	1359 (73.1)	79 (79.8)	692 (86.4)	434 (62.4)	154 (58.6)
Yes	500 (26.9)	20 (20.2)	109 (13.6)	262 (37.6)	109 (41.4)
Brain					
No	1800 (96.8)	98 (99.0)	785 (98.0)	672 (96.6)	245 (93.2)
Yes	59 (3.2)	1 (1.0)	16 (2.0)	24 (3.4)	18 (6.8)
Other					
No	1507 (81.1)	90 (90.9)	680 (84.9)	539 (77.4)	198 (75.3)
Yes	352 (18.9)	9 (9.1)	121 (15.1)	157 (22.6)	65 (24.7)
Organ sites					
S1	1108 (59.6)	99 (100.0)	702 (87.6)	188 (27.0)	119 (45.2)
S2	537 (28.9)	0	80 (10.0)	383 (55.0)	74 (28.1)
S3	182 (9.8)	0	15 (1.9)	109 (15.7)	58 (22.1)
S4	32 (1.7)	0	4 (0.5)	16 (2.3)	12 (4.6)
Visceral					
No	910 (49.0)	61 (61.6)	585 (73.0)	198 (28.4)	66 (25.1)
Yes	949 (51.0)	38 (38.4)	216 (27.0)	498 (71.6)	197 (74.9)
Bone-only					
No	1527 (82.1)	59 (59.6)	559 (69.8)	658 (94.5)	251 (95.4)
Yes	332 (17.9)	40 (40.4)	242 (30.2)	38 (5.5)	12 (4.6)
Brain-only					
No	1847 (99.4)	98 (99.0)	793 (99.0)	693 (99.6)	263 (100.0)
Yes	12 (0.6)	1 (1.0)	8 (1.0)	3 (0.4)	0

Abbreviations: ER, estrogen receptor; *ERBB2*, human epidermal growth factor receptor 2; IHC, immunohistochemistry; S1-S4, sites of metastases.

**Table 2.** Recursive Partitioning Analysis Groups

Rank	3-y Survival rate (95% CI)	Node	Patients, No.	Patient characteristics	Stage group
1	0.62 (0.56-0.69)	14	241	S1 and *ERBB2*+	B
2	0.60 (0.56-0.64)	8	485	S1, ER+, *ERBB2*−, grade 1-2, and brain-only: no/T0-3	B
3	0.52 (0.44-0.61)	9	130	S1, ER+, *ERBB2*−, grade 1-2, and brain-only: no/T4	B
4	0.50 (0.23-1.00)	13	6	S1, ER+, *ERBB2*−, grade 3, and lobular or other	B
5	0.41 (0.33-0.53)	12	93	S1, ER+, *ERBB2*−, grade 3, and ductal	C
6	0.40 (0.36-0.44)	15	537	S2	C
7	0.36 (0.30-0.44)	18	166	S3-S4 and ER+	C
8	0.17 (0.13-0.25)	4	145	S1, ER−, and *ERBB2*-	D
9	0.12 (0.02-0.78)	10	8	S1, ER+, *ERBB2*−, grade 1-2, and brain-only: yes	D
10	0.08 (0.03-0.21)	17	48	S3-S4 and ER−	D

Abbreviations: ER, estrogen receptor; *ERBB2*, human epidermal growth factor receptor 2; S1-S4, sites of metastases; −, negative; +, positive.

We found the best 3-year survival rate among patients with involvement of 1 organ site and *ERBB2*-positive disease, irrespective of ER status, followed closely by patients with ER-positive, ER-negative disease, grade 1 to 2 tumors, and no brain metastases. Generally, patients with involvement of 1 organ site had better outcomes than those with multiple sites involved. Furthermore, all patients with S1, ER-negative/*ERBB2*-negative or S3 to S4, ER-negative disease were allocated to IVd. A large group was identified among patients with S2 disease, regardless of receptor status (537 patients [28.9%]).

The number of organ sites involved was the most crucial variable for the RPA (eFigure 2 in Supplement 1). We observed a tendency to include more factors when only 1 organ site was involved. For patients with 2 organ sites, this was the only factor involved in the RPA, and for patients with 3 to 4 sites, ER status was included. For patients with 1 organ site involved, the RPA considered ER, *ERBB2*, grade, histologic findings, brain-only, and tumor size. Bone-only and visceral disease were not used in any group by the RPA. No groups could be allocated to group IVa (>70% 3-year OS rate).

**Figure 1.**
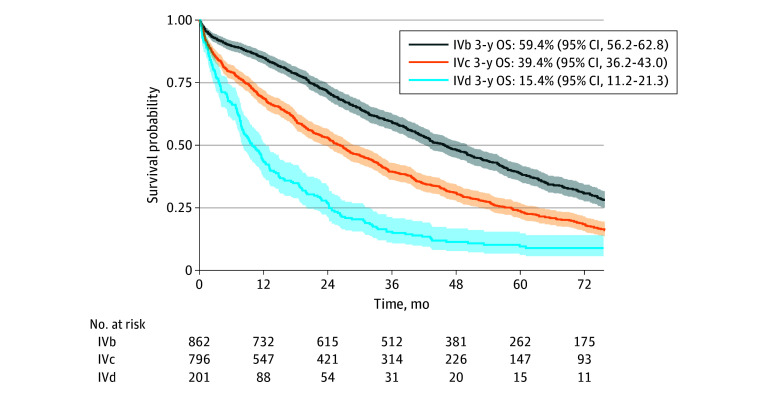
Overall Survival (OS) for Patients With de Novo Metastatic Breast Cancer 2010 to 2019 by Recursive Partitioning Assigned Stage Three-year OS rates were estimated for each terminal node, and patients were grouped based on their OS rate according to Plichta and colleagues: group IVa, less than 70%; group IVb, 50% to 70%; group IVc, 25% to less than 50%; and group IVd, less than 25%.

Figure 1 depicts unadjusted overall survival stratified by RPA-assigned stage IVb to IVd with 3-year OS rates of 59.4% (95% CI, 56.2%-62.8%) for IVb, 39.4% (95% CI, 36.2%-43.0%) for IVc, and 15.4% (95% CI, 11.2%-21.3%) for IVd (log-rank *P* < .001). eFigure 3 in Supplement 1 illustrates unadjusted OS stratified by stage and year of diagnosis to investigate potential differences between patients diagnosed in the early 2010s vs later years. No statistically significant differences were observed.

### Bootstrapping

**Figure 2.**
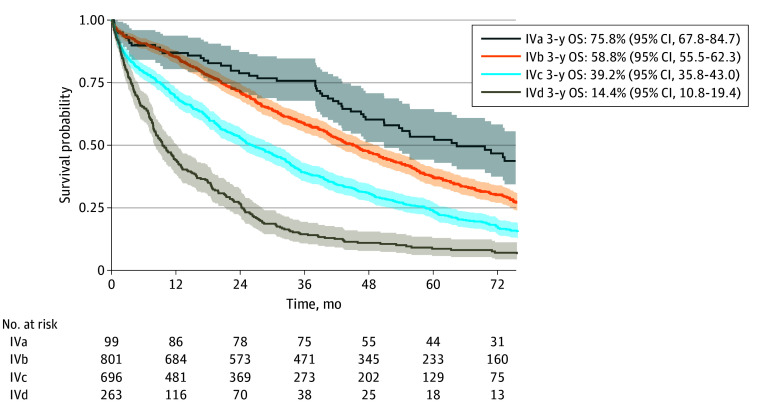
Overall Survival (OS) for Patients With de Novo Metastatic Breast Cancer 2010 to 2019 by Bootstrapping Assigned Stage Three-year OS rates were estimated for each terminal node, and patients were grouped based on their OS rate according to Plichta and colleagues: group IVa, less than 70%; group IVb, 50% to 70%; group IVc, 25% to less than 50%; and group IVd, less than 25%.

Bootstrapping was subsequently applied with 1000 iterations (eTable in Supplement 1). Out of 192 possible combinations from the RPA, 172 (including all variables) were available in our dataset. In total, 8 profiles were assigned to stage IVa, 56 to IVb, 60 to IVc, and 48 to IVd based on OS rates. Compared with the original RPA, 68 profiles were assigned a different stage after bootstrapping. This was particularly noticeable among patients with S2 disease who, according to RPA, were all assigned IVc, but with bootstrapping, 11 profiles were allocated to IVd when ER was included (ER-negative). A similar trend was observed among patients with S3 to S4, ER-negative, *ERBB2*-positive disease, who transitioned from IVd to IVc in the bootstrap analysis. Table 3 demonstrates how patients shifted from RPA to bootstrap stage assignment. Figure 2 displays unadjusted OS by assigned bootstrap stage for all patients (3-year OS rates: IVa, 75.8%; 95% CI, 67.8%-84.7%; IVb, 58.8%; 95% CI, 55.5%-62.3%; IVc, 39.2%; 95% CI, 35.8%-43.0%; and IVd, 14.4%; 95% CI, 10.8%-19.4%; *P* < .001) and by year of diagnosis (eFigure 4 in Supplement 1).

**Table 3.** Distribution Between Recursive Partitioning Analysis (RPA) and Bootstrap Stage Assignment

Stage by RPA	Stage by bootstrap			
	IVa	IVb	IVc	IVd
IVb	99 (11.5)	643 (74.6)	120 (13.9)	0
IVc	0	153 (19.2)	531 (66.7)	112 (14.1)
IVd	0	5 (2.5)	45 (22.4)	151 (75.1)

## Discussion

We successfully assigned a nationwide cohort of patients with dnMBC to clinically relevant and prognostically distinct subgroups, thereby independently validating the methods of a recent prognostic model for dnMBC proposed by Plichta and colleagues, even with fewer clinical factors used.^3^ This prognostic model could serve as a stratification factor in clinical trials involving patients with dnMBC and might influence future revisions of the current AJCC staging guidelines.

We observed a decrease in prognosis with an increasing number of involved organ sites and ER negativity. Regardless of other characteristics, patients with 3 or 4 organ sites remained in the poorest prognostic groups. Using RPA, we were thus able to decipher the heterogeneity of dnMBC.^3^

Despite our study encompassing 1859 patients, we managed to replicate the methods of the Plichta system, demonstrating the robustness of the RPA analysis and bootstrapping even in a modified dataset with fewer variables. However, we could not identify a group with a 3-year survival rate of over 70%, corresponding to stage IVa by RPA, but bootstrapping assigned 8 patient characteristic profiles to IVa. This limitation in the RPA might be due to a small sample size.

Upon considering the 2 patient characteristic profiles assigned IVa in the Plichta system, our bootstrapping (eTable in Supplement 1) allocated a mix of stage IVa and IVb. This suggests the difficulty in identifying a true IVa group with a favorable outcome based solely on clinical and pathological features, indicating the need for further investigations, possibly involving tumor genomic examinations.

Our RPA identified 10 different subgroups with 3-year survival rates varying from 62% to 8%, using 7 of 9 available variables. An intriguing observation from bootstrapping was that nearly any patient diagnosed with an ER- and *ERBB2*-negative disease would be allocated to stage IVd. This underscores the necessity for improved treatment options for these patients, especially with the potential impact of checkpoint immunotherapy on outcomes. Hence, validating the robustness of the method in a new treatment landscape for dnMBC is essential.^9^

The Plichta system represents an improvement over the current clinical practice prognostic scoring, primarily incorporating immunohistochemical subtypes for dnMBC. It offers crucial additional information for clinicians to identify patients with particularly poor or good prognoses up front, which could be particularly significant for patients with luminal or *ERBB2*-positive tumors. The subgroups identified in the Plichta system might reclassify them into stage IVc or IVd.

If implemented as part of the AJCC staging system, the Plichta system could be used as a common system and facilitate the development of an easily accessible online tool. This tool would allow clinicians to retrieve a further subdivided stage IV grouping based on specific clinical factors entered. It might be necessary to accommodate for missing prognostic information such as PR status. However, we have shown that even a modified system without PR is still able to identify 4 prognostic groups for stage IV disease, which may improve the wider uptake of the system. Others might even find that the system can be simplified even further. The lack of PR status in our data meant that we could not apply the Plichta system without revising the classification.

Staging dnMBC using RPA represents a novel approach distinct from models based on regression analysis, such as PREDICT for prognostication in early breast cancer.^10^ While logistic regression could have been applied, the strength of RPA lies in its ease of translation to clinical practice and its visual representation of the hierarchy of involved clinical factors.

### Strengths and Limitations

Study strengths include the national, population-based registry of the DBCG. Additionally, we have complete data for almost all patients, and patient inclusion in the database is not contingent upon treatment initiation.

Limitations include reduced power in the RPA due to our patient numbers (as evident in the end-node of S2 patients and missing IVa subgroup), lack of information on PR status (which is included in the Plichta system), and the retrospective nature of our study, potentially introducing confirmation bias. Furthermore, the performance of our system was assessed in the same dataset in which it was developed, which may overestimate the assessment of performance. However, this was partly compensated by the bootstrapping which acts as an internal validation and was also applied by Plichta.

## Conclusions

Our findings offer external and independent validation of the methods applied in the novel Plichta staging system for dnMBC. We have demonstrated that recursive partitioning and bootstrapping can stratify patients with dnMBC into 4 different prognostic groups by estimated outcome and that future staging of dnMBC should take these factors into account. Furthermore, we have shown that fewer variables than originally applied in the Plichta system remain effective in distinguishing these patients and may expand the uptake of the staging system.

## Notes

Supplement 1. **eFigure 1. **Inclusion Diagram **eFigure 2. **Recursive Partitioning Analysis Decision Tree **eFigure 3. **Overall Survival by Recursive Partitioning Assigned Stage and Year of Diagnosis **eFigure 4. **Overall Survival by Bootstrapping Assigned Stage and Year of Diagnosis **eTable. **Bootstrapping Results by Characteristic Profile

Supplement 2.
Data Sharing Statement
