# Supplementary material for: Development and Methodological Validation of a Modified Staging System for de Novo Metastatic Breast Cancer
Source: JAMA Netw Open. 2024 Mar 13;7(3):e242174. doi: 10.1001/jamanetworkopen.2024.2174 (PMC10938173; doi:10.1001/jamanetworkopen.2024.2174)
Supplement: Supplement 1. — eFigure 1. Inclusion Diagram eFigure 2. Recursive Partitioning Analysis Decision Tree eFigure 3. Overall Survival by Recursive Partitioning Assigned Stage and Year of Diagnosis eFigure 4. Overall Survival by Bootstrapping Assigned Stage and Year of Diagnosis eTable. Bootstrapping Results by Characteristic Profile [file jamanetwopen-e242174-s001.pdf]

Supplemental Online Content

Berg T, Jensen M-B, Rossing M, et al. Development and external validation of a modified staging system for de novo metastatic breast cancer. *JAMA Netw Open*. 2024;7(3):e242174. doi:10.1001/jamanetworkopen.2024.2174

eFigure 1. Inclusion Diagram

eFigure 2. Recursive Partitioning Analysis Decision Tree

eFigure 3. Overall Survival by Recursive Partitioning Assigned Stage and Year of Diagnosis

eFigure 4. Overall Survival by Bootstrapping Assigned Stage and Year of Diagnosis

eTable. Bootstrapping Results by Characteristic Profile

This supplemental material has been provided by the authors to give readers additional information about their work.

© 2024 Berg T et al. *JAMA Network Open.*

eFigure 1. Inclusion Diagram

dnMBC: de novo metastatic breast cancer, HER2: human epidermal growth factor receptor 2

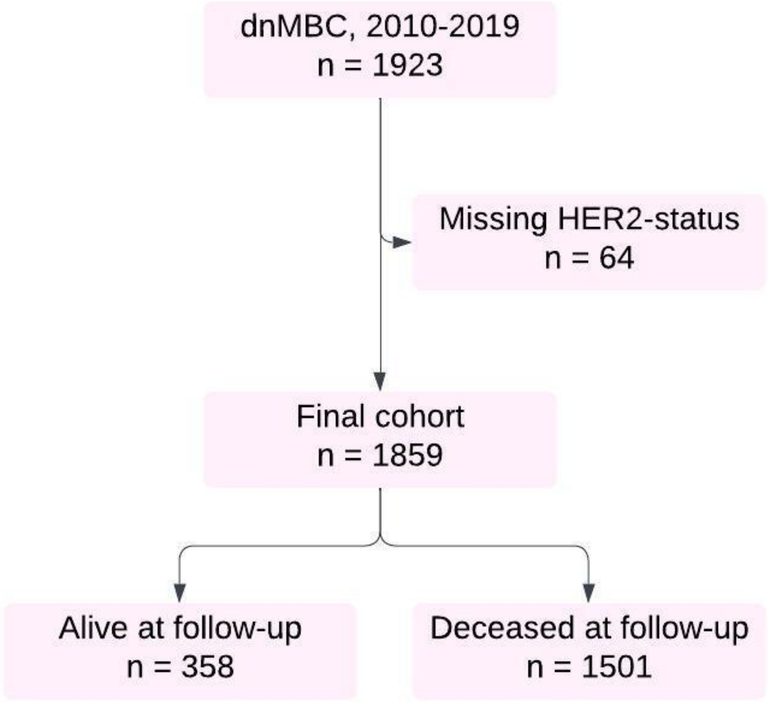

eFigure 2. Recursive Partitioning Analysis Decision Tree

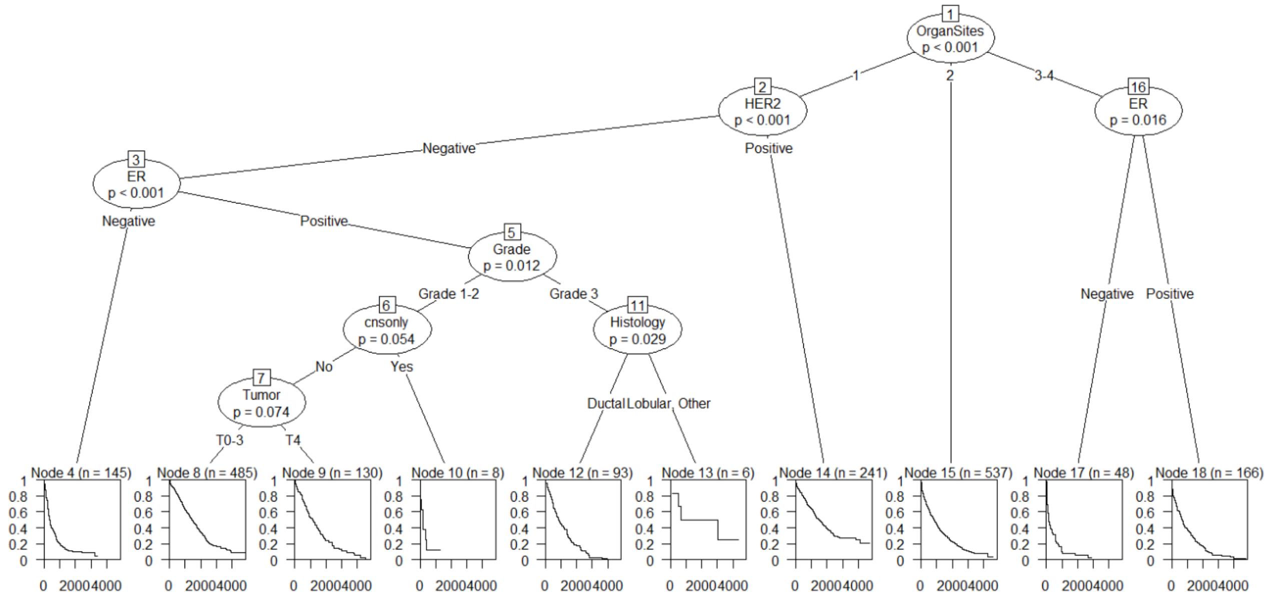

© 2024 Berg T et al. *JAMA Network Open.*

eFigure 3. Overall Survival by Recursive Partitioning Assigned Stage and Year of Diagnosis. IVb 50% to 70%, IVc, 25 to <50% and IVd <25%.

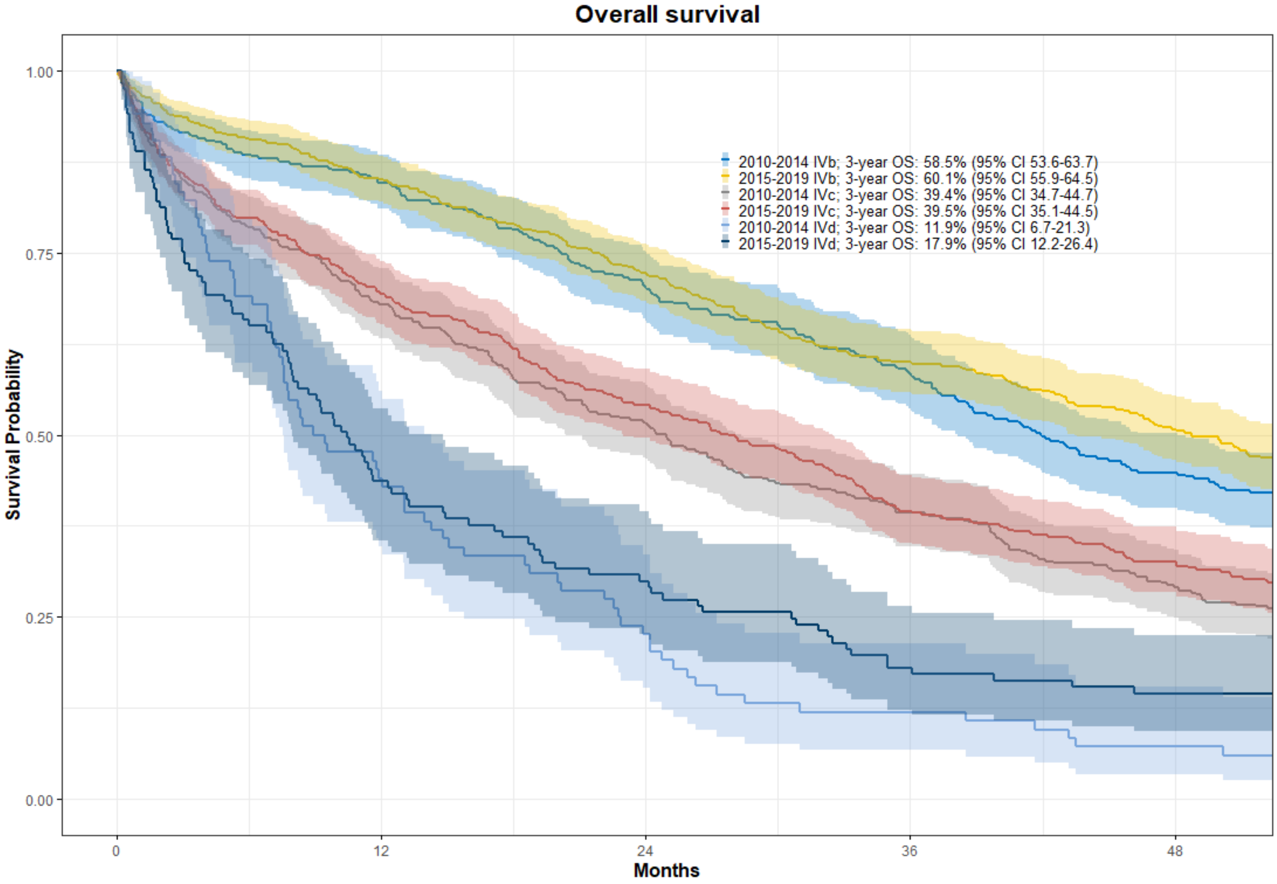

Number at risk

366	310	257	214	164
496	422	358	298	217
371	252	191	146	108
425	295	230	168	118
84	37	19	10	6
117	51	35	21	14

© 2024 Berg T et al. *JAMA Network Open.*

eFigure 4. Overall Survival by Bootstrapping Assigned Stage and Year of Diagnosis. IVa > 70%, IVb 50% to 70%, IVc, 25 to <50% and IVd <25%.

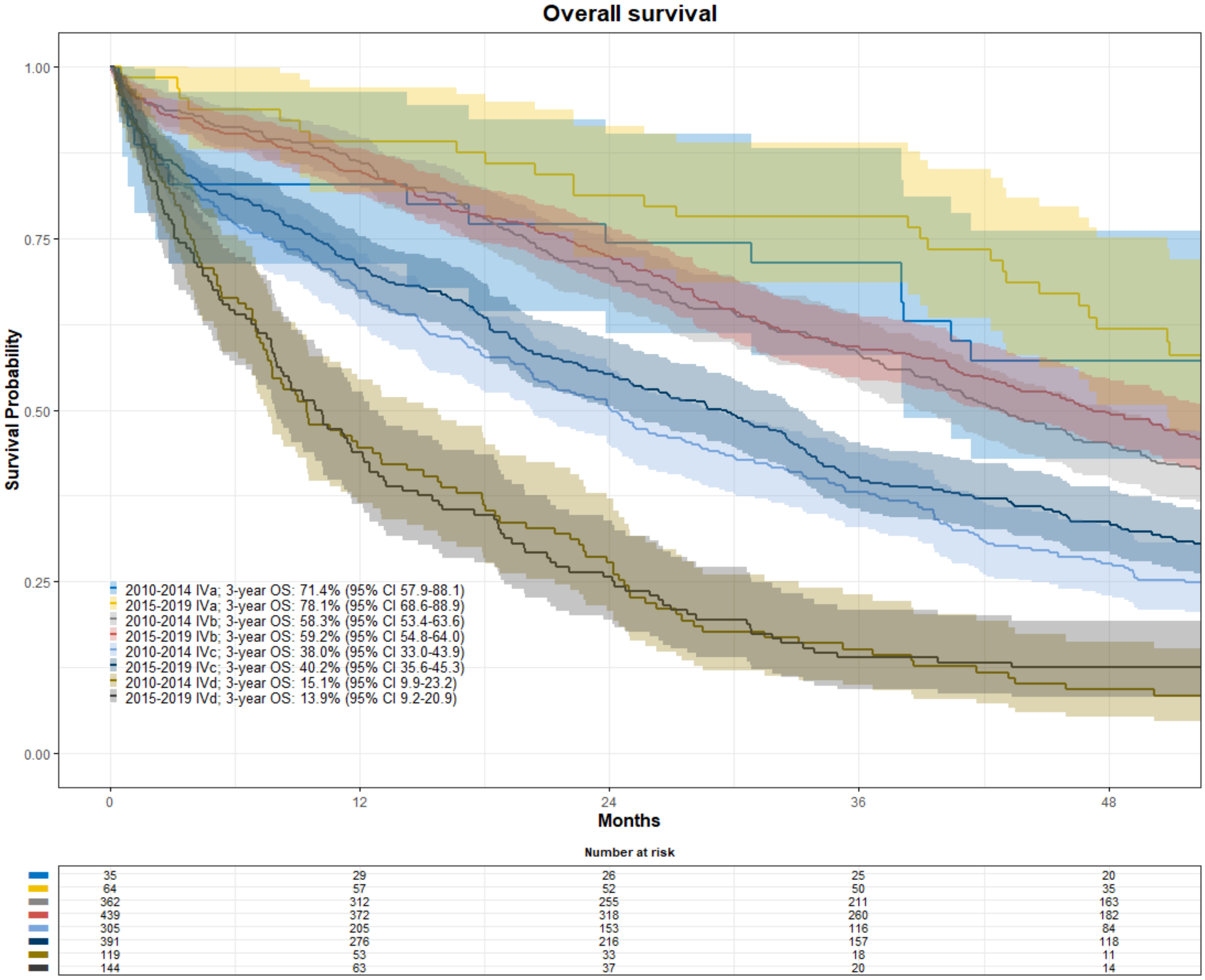

© 2024 Berg T et al. *JAMA Network Open.*

eTable. Bootstrapping Results by Characteristic Profile

Green indicates most commonly occurring group made by >60% of all models, yellow indicates most commonly occurring group in <60% of all models. Red indicates characteristic profiles that changed final stage compared to the original recursive partitioning analysis (RPA).

**Characteristic Profile**	**Patients (n=1859)**	**IVa**	**IVb**	**IVc**	**IVd**	**Stage from orginal RPA**
S1/ER-/HER2-/Grade 1-2/Ductal/T0-3/Bone-Only: No/Brain-Only: No/Visceral: No	24 (1.3%)				1000 (100%)	IVd
S1/ER-/HER2-/Grade 1-2/Ductal/T0-3/Bone-Only: No/Brain-Only: No/Visceral: Yes	14 (0,8%)				1000 (100%)	IVd
S1/ER-/HER2-/Grade 1-2/Ductal/T0-3/Bone-Only: Yes/Brain-Only: No/Visceral: No	7 (0,4%)			206 (20,6%)	794 (79,4%)	IVd
S1/ER-/HER2-/Grade 1-2/Ductal/T4/Bone-Only: No/Brain-Only: No/Visceral: No	7 (0,4%)			17 (1,7%)	983 (98,3%)	IVd
S1/ER-/HER2-/Grade 1-2/Ductal/T4/Bone-Only: No/Brain-Only: No/Visceral: Yes	7 (0,4%)		5 (0,5%)	16 (1,6%)	979 (97,9%)	IVd
S1/ER-/HER2-/Grade 1-2/Ductal/T4/Bone-Only: Yes/Brain-Only: No/Visceral: No	2 (0,1%)			28 (2,8%)	972 (97,2%)	IVd
S1/ER-/HER2-/Grade 1-2/Lobular/T0-3/Bone-Only: No/Brain-Only: No/Visceral: No	1 (0,1%)		4 (0,4%)	37 (3,7%)	959 (95,9%)	IVd
S1/ER-/HER2-/Grade 1-2/Lobular/T0-3/Bone-Only: No/Brain-Only: No/Visceral: Yes	1 (0,1%)		3 (0,3%)	4 (0,4%)	993 (99,3%)	IVd
S1/ER-/HER2-/Grade 1-2/Lobular/T0-3/Bone-Only: Yes/Brain-Only: No/Visceral: No	2 (0,1%)		6 (0,6%)	241 (24,1%)	753 (75,3%)	IVd
S1/ER-/HER2-/Grade 1-2/Other/T0-3/Bone-Only: No/Brain-Only: No/Visceral: No	5 (0,3%)			35 (3,5%)	965 (96,5%)	IVd
S1/ER-/HER2-/Grade 1-2/Other/T0-3/Bone-Only: No/Brain-Only: No/Visceral: Yes	2 (0,1%)			1 (0,1%)	999 (99,9%)	IVd
S1/ER-/HER2-/Grade 1-2/Other/T4/Bone-Only: No/Brain-Only: No/Visceral: No	1 (0,1%)		37 (3,7%)	9 (0,9%)	954 (95,4%)	IVd
S1/ER-/HER2-/Grade 1-2/Other/T4/Bone-Only: No/Brain-Only: No/Visceral: Yes	2 (0,1%)		36 (3,6%)	11 (1,1%)	953 (95,3%)	IVd
S1/ER-/HER2-/Grade 3/Ductal/T0-3/Bone-Only: No/Brain-Only: No/Visceral: No	19 (1,0%)			887 (88,7%)	113 (11,3%)	IVd
S1/ER-/HER2-/Grade 3/Ductal/T0-3/Bone-Only: No/Brain-Only: No/Visceral: Yes	22 (1,2%)			1 (0,1%)	999 (99,9%)	IVd
S1/ER-/HER2-/Grade 3/Ductal/T0-3/Bone-Only: Yes/Brain-Only: No/Visceral: No	6 (0,3%)		3 (0,3%)	800 (80%)	197 (19,7%)	IVd
S1/ER-/HER2-/Grade 3/Ductal/T4/Bone-Only: No/Brain-Only: No/Visceral: No	10 (0,5%)			14 (1,4%)	986 (98,6%)	IVd
S1/ER-/HER2-/Grade 3/Ductal/T4/Bone-Only: No/Brain-Only: No/Visceral: Yes	9 (0,5%)			15 (1,5%)	985 (98,5%)	IVd
S1/ER-/HER2-/Grade 3/Ductal/T4/Bone-Only: Yes/Brain-Only: No/Visceral: No	1 (0,1%)			19 (1,9%)	981 (98,1%)	IVd
S1/ER-/HER2-/Grade 3/Lobular/T0-3/Bone-Only: No/Brain-Only: No/Visceral: No	1 (0,1%)			707 (70,7%)	293 (29,3%)	IVd
S1/ER-/HER2-/Grade 3/Other/T0-3/Bone-Only: No/Brain-Only: No/Visceral: Yes	2 (0,1%)			4 (0,4%)	996 (99,6%)	IVd
S1/ER-/HER2+/Grade 1-2/Ductal/T0-3/Bone-Only: No/Brain-Only: No/Visceral: No	9 (0,5%)	866 (86,6%)	134 (13,4%)			IVb
S1/ER-/HER2+/Grade 1-2/Ductal/T0-3/Bone-Only: No/Brain-Only: No/Visceral: Yes	17 (1,0%)	576 (57,6%)	424 (42,4%)			IVb

© 2024 Berg T et al. *JAMA Network Open.*

Characteristic Profile	Patients (n=1859)	IVa	IVb	IVc	IVd	Stage from orginal RPA
S1/ER-/HER2+/Grade 1-2/Ductal/T0-3/Bone-Only: Yes/Brain-Only: No/Visceral: No	8 (0,4%)	546 (54,6%)	454 (45,4%)			IVb
S1/ER-/HER2+/Grade 1-2/Ductal/T4/Bone-Only: No/Brain-Only: No/Visceral: No	9 (0,5%)	315 (31,5%)	658 (65,8%)	27 (2,7%)		IVb
S1/ER-/HER2+/Grade 1-2/Ductal/T4/Bone-Only: No/Brain-Only: No/Visceral: Yes	6 (0,3%)	267 (26,7%)	619 (61,9%)	114 (11,4%)		IVb
S1/ER-/HER2+/Grade 1-2/Lobular/T0-3/Bone-Only: No/Brain-Only: No/Visceral: No	1 (0,1%)	228 (22,8%)	570 (57%)	112 (11,2%)	90 (9%)	IVb
S1/ER-/HER2+/Grade 1-2/Lobular/T0-3/Bone-Only: No/Brain-Only: No/Visceral: Yes	1 (0,1%)	37 (3,7%)	746 (74,6%)	123 (12,3%)	94 (9,4%)	IVb
S1/ER-/HER2+/Grade 1-2/Other/T0-3/Bone-Only: No/Brain-Only: No/Visceral: Yes	4 (0,2%)	62 (6,2%)	730 (73%)	114 (11,4%)	94 (9,4%)	IVb
S1/ER-/HER2+/Grade 1-2/Other/T4/Bone-Only: No/Brain-Only: No/Visceral: Yes	1 (0,1%)	8 (0,8%)	794 (79,4%)	106 (10,6%)	92 (9,2%)	IVb
S1/ER-/HER2+/Grade 3/Ductal/T0-3/Bone-Only: No/Brain-Only: No/Visceral: No	11 (0,6%)	678 (67,8%)	322 (32,2%)			IVb
S1/ER-/HER2+/Grade 3/Ductal/T0-3/Bone-Only: No/Brain-Only: No/Visceral: Yes	22 (1,2%)	57 (5,7%)	943 (94,3%)			IVb
S1/ER-/HER2+/Grade 3/Ductal/T0-3/Bone-Only: No/Brain-Only: Yes/Visceral: No	1 (0,1%)	678 (67,8%)	322 (32,2%)			IVb
S1/ER-/HER2+/Grade 3/Ductal/T0-3/Bone-Only: Yes/Brain-Only: No/Visceral: No	4 (0,2%)	106 (10,6%)	559 (55,9%)	335 (33,5%)		IVb
S1/ER-/HER2+/Grade 3/Ductal/T4/Bone-Only: No/Brain-Only: No/Visceral: No	6 (0,3%)	11 (1,1%)	376 (37,6%)	606 (60,6%)	7 (0,7%)	IVb
S1/ER-/HER2+/Grade 3/Ductal/T4/Bone-Only: No/Brain-Only: No/Visceral: Yes	10 (0,5%)		293 (29,3%)	701 (70,1%)	6 (0,6%)	IVb
S1/ER-/HER2+/Grade 3/Lobular/T0-3/Bone-Only: No/Brain-Only: No/Visceral: No	1 (0,1%)	144 (14,4%)	658 (65,8%)	108 (10,8%)	90 (9%)	IVb
S1/ER-/HER2+/Grade 3/Lobular/T0-3/Bone-Only: No/Brain-Only: Yes/Visceral: No	1 (0,1%)	144 (14,4%)	658 (65,8%)	108 (10,8%)	90 (9%)	IVb
S1/ER-/HER2+/Grade 3/Lobular/T4/Bone-Only: No/Brain-Only: No/Visceral: No	1 (0,1%)	8 (0,8%)	544 (54,4%)	351 (35,1%)	97 (9,7%)	IVb
S1/ER+/HER2-/Grade 1-2/Ductal/T0-3/Bone-Only: No/Brain-Only: No/Visceral: No	139 (7,5%)		1000 (100%)			IVb
S1/ER+/HER2-/Grade 1-2/Ductal/T0-3/Bone-Only: No/Brain-Only: No/Visceral: Yes	54 (2,9%)		1000 (100%)			IVb
S1/ER+/HER2-/Grade 1-2/Ductal/T0-3/Bone-Only: No/Brain-Only: Yes/Visceral: No	4 (0,2%)		914 (91,4%)		86 (8,6%)	IVd
S1/ER+/HER2-/Grade 1-2/Ductal/T0-3/Bone-Only: Yes/Brain-Only: No/Visceral: No	134 (7,2%)		1000 (100%)			IVb
S1/ER+/HER2-/Grade 1-2/Ductal/T4/Bone-Only: No/Brain-Only: No/Visceral: No	52 (2,8%)		213 (21,3%)	787 (78,7%)		IVb
S1/ER+/HER2-/Grade 1-2/Ductal/T4/Bone-Only: No/Brain-Only: No/Visceral: Yes	20 (1,1%)		967 (96,7%)	33 (3,3%)		IVb
S1/ER+/HER2-/Grade 1-2/Ductal/T4/Bone-Only: No/Brain-Only: Yes/Visceral: No	3 (0,2%)		128 (12,8%)	786 (78,6%)	86 (8,6%)	IVd
S1/ER+/HER2-/Grade 1-2/Ductal/T4/Bone-Only: Yes/Brain-Only: No/Visceral: No	21 (1,1%)		29 (2,9%)	971 (97,1%)		IVb
S1/ER+/HER2-/Grade 1-2/Lobular/T0-3/Bone-Only: No/Brain-Only: No/Visceral: No	43 (2,3%)		997 (99,7%)	3 (0,3%)		IVb
S1/ER+/HER2-/Grade 1-2/Lobular/T0-3/Bone-Only: No/Brain-Only: No/Visceral: Yes	6 (0,3%)		990 (99%)	10 (1%)		IVb

© 2024 Berg T et al. *JAMA Network Open.*

Characteristic Profile	Patients (n=1859)	IVa	IVb	IVc	IVd	Stage from orginal RPA
S1/ER+/HER2-/Grade 1-2/Lobular/T0-3/Bone-Only: Yes/Brain-Only: No/Visceral: No	61 (3,3%)		1000 (100%)			IVb
S1/ER+/HER2-/Grade 1-2/Lobular/T4/Bone-Only: No/Brain-Only: No/Visceral: No	17 (0,9%)		552 (55,2%)	448 (44,8%)		IVb
S1/ER+/HER2-/Grade 1-2/Lobular/T4/Bone-Only: No/Brain-Only: No/Visceral: Yes	2 (0,1%)		796 (79,6%)	204 (20,4%)		IVb
S1/ER+/HER2-/Grade 1-2/Lobular/T4/Bone-Only: Yes/Brain-Only: No/Visceral: No	7 (0,4%)	16 (1,6%)	601 (60,1%)	383 (38,3%)		IVb
S1/ER+/HER2-/Grade 1-2/Other/T0-3/Bone-Only: No/Brain-Only: No/Visceral: No	19 (1,0%)		1000 (100%)			IVb
S1/ER+/HER2-/Grade 1-2/Other/T0-3/Bone-Only: No/Brain-Only: No/Visceral: Yes	11 (0,6%)		988 (98,8%)	12 (1,2%)		IVb
S1/ER+/HER2-/Grade 1-2/Other/T0-3/Bone-Only: No/Brain-Only: Yes/Visceral: No	1 (0,1%)		916 (91,6%)		84 (8,4%)	IVd
S1/ER+/HER2-/Grade 1-2/Other/T0-3/Bone-Only: Yes/Brain-Only: No/Visceral: No	18 (1,0%)	782 (78,2%)	218 (21,8%)			IVb
S1/ER+/HER2-/Grade 1-2/Other/T4/Bone-Only: No/Brain-Only: No/Visceral: No	9 (0,5%)	456 (45,6%)	492 (49,2%)	52 (5,2%)		IVb
S1/ER+/HER2-/Grade 1-2/Other/T4/Bone-Only: No/Brain-Only: No/Visceral: Yes	1 (0,1%)	380 (38%)	596 (59,6%)	24 (2,4%)		IVb
S1/ER+/HER2-/Grade 1-2/Other/T4/Bone-Only: Yes/Brain-Only: No/Visceral: No	1 (0,1%)	346 (34,6%)	379 (37,9%)	275 (27,5%)		IVb
S1/ER+/HER2-/Grade 3/Ductal/T0-3/Bone-Only: No/Brain-Only: No/Visceral: No	30 (1,6%)		884 (88,4%)	116 (11,6%)		IVc
S1/ER+/HER2-/Grade 3/Ductal/T0-3/Bone-Only: No/Brain-Only: No/Visceral: Yes	18 (1,0%)		4 (0,4%)	996 (99,6%)		IVc
S1/ER+/HER2-/Grade 3/Ductal/T0-3/Bone-Only: Yes/Brain-Only: No/Visceral: No	24 (1,3%)		655 (65,5%)	345 (34,5%)		IVc
S1/ER+/HER2-/Grade 3/Ductal/T4/Bone-Only: No/Brain-Only: No/Visceral: No	10 (0,5%)		7 (0,7%)	693 (69,3%)	300 (30%)	IVc
S1/ER+/HER2-/Grade 3/Ductal/T4/Bone-Only: No/Brain-Only: No/Visceral: Yes	5 (0,3%)		130 (13%)	751 (75,1%)	119 (11,9%)	IVc
S1/ER+/HER2-/Grade 3/Ductal/T4/Bone-Only: Yes/Brain-Only: No/Visceral: No	6 (0,3%)			693 (69,3%)	307 (30,7%)	IVc
S1/ER+/HER2-/Grade 3/Lobular/T0-3/Bone-Only: Yes/Brain-Only: No/Visceral: No	2 (0,1%)		659 (65,9%)	341 (34,1%)		IVb
S1/ER+/HER2-/Grade 3/Lobular/T4/Bone-Only: No/Brain-Only: No/Visceral: No	1 (0,1%)		184 (18,4%)	652 (65,2%)	164 (16,4%)	IVb
S1/ER+/HER2-/Grade 3/Other/T0-3/Bone-Only: No/Brain-Only: No/Visceral: Yes	1 (0,1%)		88 (8,8%)	912 (91,2%)	0 (0%)	IVb
S1/ER+/HER2-/Grade 3/Other/T0-3/Bone-Only: Yes/Brain-Only: No/Visceral: No	1 (0,1%)		667 (66,7%)	333 (33,3%)	0 (0%)	IVb
S1/ER+/HER2-/Grade 3/Other/T4/Bone-Only: No/Brain-Only: No/Visceral: Yes	1 (0,1%)	148 (14,8%)	193 (19,3%)	652 (65,2%)	7 (0,7%)	IVb
S1/ER+/HER2+/Grade 1-2/Ductal/T0-3/Bone-Only: No/Brain-Only: No/Visceral: No	24 (1,3%)	246 (24,6%)	754 (75,4%)			IVb
S1/ER+/HER2+/Grade 1-2/Ductal/T0-3/Bone-Only: No/Brain-Only: No/Visceral: Yes	21 (1,1%)	601 (60,1%)	399 (39,9%)			IVb
S1/ER+/HER2+/Grade 1-2/Ductal/T0-3/Bone-Only: No/Brain-Only: Yes/Visceral: No	1 (0,1%)	242 (24,2%)	743 (74,3%)		15 (1,5%)	IVb
S1/ER+/HER2+/Grade 1-2/Ductal/T0-3/Bone-Only: Yes/Brain-Only: No/Visceral: No	14 (0,8%)	539 (53,9%)	461 (46,1%)			IVb

© 2024 Berg T et al. *JAMA Network Open.*

Characteristic Profile	Patients (n=1859)	IVa	IVb	IVc	IVd	Stage from orginal RPA
S1/ER+/HER2+/Grade 1-2/Ductal/T4/Bone-Only: No/Brain-Only: No/Visceral: No	12 (0,6%)		299 (29,9%)	701 (70,1%)		IVb
S1/ER+/HER2+/Grade 1-2/Ductal/T4/Bone-Only: No/Brain-Only: No/Visceral: Yes	6 (0,3%)		157 (15,7%)	754 (75,4%)	89 (8,9%)	IVb
S1/ER+/HER2+/Grade 1-2/Ductal/T4/Bone-Only: Yes/Brain-Only: No/Visceral: No	3 (0,2%)	226 (22,6%)	263 (26,3%)	511 (51,1%)		IVb
S1/ER+/HER2+/Grade 1-2/Lobular/T0-3/Bone-Only: No/Brain-Only: No/Visceral: No	3 (0,2%)	225 (22,5%)	670 (67%)	105 (10,5%)		IVb
S1/ER+/HER2+/Grade 1-2/Lobular/T0-3/Bone-Only: No/Brain-Only: No/Visceral: Yes	1 (0,1%)	32 (3,2%)	845 (84,5%)	119 (11,9%)	4 (0,4%)	IVb
S1/ER+/HER2+/Grade 1-2/Lobular/T0-3/Bone-Only: Yes/Brain-Only: No/Visceral: No	1 (0,1%)	288 (28,8%)	609 (60,9%)	103 (10,3%)		IVb
S1/ER+/HER2+/Grade 1-2/Other/T0-3/Bone-Only: No/Brain-Only: No/Visceral: No	2 (0,1%)	241 (24,1%)	662 (66,2%)	97 (9,7%)		IVb
S1/ER+/HER2+/Grade 1-2/Other/T4/Bone-Only: No/Brain-Only: No/Visceral: No	2 (0,1%)	77 (7,7%)	544 (54,4%)	379 (37,9%)		IVb
S1/ER+/HER2+/Grade 3/Ductal/T0-3/Bone-Only: No/Brain-Only: No/Visceral: No	7 (0,4%)	463 (46,3%)	534 (53,4%)	3 (0,3%)		IVb
S1/ER+/HER2+/Grade 3/Ductal/T0-3/Bone-Only: No/Brain-Only: No/Visceral: Yes	13 (0,7%)	316 (31,6%)	683 (68,3%)	1 (0,1%)		IVb
S1/ER+/HER2+/Grade 3/Ductal/T0-3/Bone-Only: No/Brain-Only: Yes/Visceral: No	1 (0,1%)	463 (46,3%)	531 (53,1%)	3 (0,3%)	3 (0,3%)	IVb
S1/ER+/HER2+/Grade 3/Ductal/T0-3/Bone-Only: Yes/Brain-Only: No/Visceral: No	7 (0,4%)		614 (61,4%)	377 (37,7%)	9 (0,9%)	IVb
S1/ER+/HER2+/Grade 3/Ductal/T4/Bone-Only: No/Brain-Only: No/Visceral: No	2 (0,1%)	37 (3,7%)	291 (29,1%)	662 (66,2%)	10 (1%)	IVb
S1/ER+/HER2+/Grade 3/Ductal/T4/Bone-Only: No/Brain-Only: No/Visceral: Yes	3 (0,2%)		187 (18,7%)	716 (71,6%)	97 (9,7%)	IVb
S1/ER+/HER2+/Grade 3/Ductal/T4/Bone-Only: Yes/Brain-Only: No/Visceral: No	2 (0,1%)		248 (24,8%)	708 (70,8%)	44 (4,4%)	IVb
S1/ER+/HER2+/Grade 3/Lobular/T4/Bone-Only: No/Brain-Only: No/Visceral: No	3 (0,2%)	38 (3,8%)	562 (56,2%)	399 (39,9%)	1 (0,1%)	IVb
S2/ER-/HER2-/Grade 1-2/Ductal/T0-3/Bone-Only: No/Brain-Only: No/Visceral: Yes	16 (0,9%)			376 (37,6%)	624 (62,4%)	IVc
S2/ER-/HER2-/Grade 1-2/Ductal/T4/Bone-Only: No/Brain-Only: No/Visceral: Yes	3 (0,2%)			286 (28,6%)	714 (71,4%)	IVc
S2/ER-/HER2-/Grade 1-2/Lobular/T0-3/Bone-Only: No/Brain-Only: No/Visceral: No	1 (0,1%)			306 (30,6%)	694 (69,4%)	IVc
S2/ER-/HER2-/Grade 1-2/Lobular/T4/Bone-Only: No/Brain-Only: No/Visceral: Yes	1 (0,1%)			289 (28,9%)	711 (71,1%)	IVc
S2/ER-/HER2-/Grade 1-2/Other/T0-3/Bone-Only: No/Brain-Only: No/Visceral: No	1 (0,1%)		16 (1,6%)	284 (28,4%)	700 (70%)	IVc
S2/ER-/HER2-/Grade 1-2/Other/T0-3/Bone-Only: No/Brain-Only: No/Visceral: Yes	2 (0,1%)		15 (1,5%)	356 (35,6%)	629 (62,9%)	IVc
S2/ER-/HER2-/Grade 3/Ductal/T0-3/Bone-Only: No/Brain-Only: No/Visceral: No	1 (0,1%)		9 (0,9%)	136 (13,6%)	855 (85,5%)	IVc
S2/ER-/HER2-/Grade 3/Ductal/T0-3/Bone-Only: No/Brain-Only: No/Visceral: Yes	17 (0,9%)			12 (1,2%)	988 (98,8%)	IVc
S2/ER-/HER2-/Grade 3/Ductal/T4/Bone-Only: No/Brain-Only: No/Visceral: No	1 (0,1%)			17 (1,7%)	983 (98,3%)	IVc
S2/ER-/HER2-/Grade 3/Ductal/T4/Bone-Only: No/Brain-Only: No/Visceral: Yes	5 (0,3%)			48 (4,8%)	952 (95,2%)	IVc

© 2024 Berg T et al. *JAMA Network Open.*

Characteristic Profile	Patients (n=1859)	IVa	IVb	IVc	IVd	Stage from orginal RPA
S2/ER-/HER2-/Grade 3/Lobular/T0-3/Bone-Only: No/Brain-Only: No/Visceral: Yes	1 (0,1%)			75 (7,5%)	925 (92,5%)	IVc
S2/ER-/HER2+/Grade 1-2/Ductal/T0-3/Bone-Only: No/Brain-Only: No/Visceral: No	1 (0,1%)	16 (1,6%)	62 (6,2%)	416 (41,6%)	506 (50,6%)	IVc
S2/ER-/HER2+/Grade 1-2/Ductal/T0-3/Bone-Only: No/Brain-Only: No/Visceral: Yes	22 (1,2%)			278 (27,8%)	722 (72,2%)	IVc
S2/ER-/HER2+/Grade 1-2/Ductal/T4/Bone-Only: No/Brain-Only: No/Visceral: Yes	6 (0,3%)		18 (1,8%)	524 (52,4%)	458 (45,8%)	IVc
S2/ER-/HER2+/Grade 1-2/Lobular/T0-3/Bone-Only: No/Brain-Only: No/Visceral: No	1 (0,1%)	17 (1,7%)	72 (7,2%)	487 (48,7%)	424 (42,4%)	IVc
S2/ER-/HER2+/Grade 1-2/Lobular/T0-3/Bone-Only: No/Brain-Only: No/Visceral: Yes	1 (0,1%)		8 (0,8%)	361 (36,1%)	631 (63,1%)	IVc
S2/ER-/HER2+/Grade 1-2/Other/T0-3/Bone-Only: No/Brain-Only: No/Visceral: Yes	1 (0,1%)		10 (1%)	334 (33,4%)	656 (65,6%)	IVc
S2/ER-/HER2+/Grade 3/Ductal/T0-3/Bone-Only: No/Brain-Only: No/Visceral: No	2 (0,1%)	2 (0,2%)	895 (89,5%)	88 (8,8%)	15 (1,5%)	IVc
S2/ER-/HER2+/Grade 3/Ductal/T0-3/Bone-Only: No/Brain-Only: No/Visceral: Yes	22 (1,2%)	11 (1,1%)	947 (94,7%)	42 (4,2%)		IVc
S2/ER-/HER2+/Grade 3/Ductal/T4/Bone-Only: No/Brain-Only: No/Visceral: No	1 (0,1%)		505 (50,5%)	296 (29,6%)	199 (19,9%)	IVc
S2/ER-/HER2+/Grade 3/Ductal/T4/Bone-Only: No/Brain-Only: No/Visceral: Yes	4 (0,2%)		534 (53,4%)	323 (32,3%)	143 (14,3%)	IVc
S2/ER+/HER2-/Grade 1-2/Ductal/T0-3/Bone-Only: No/Brain-Only: No/Visceral: No	16 (0,9%)		918 (91,8%)	82 (8,2%)		IVc
S2/ER+/HER2-/Grade 1-2/Ductal/T0-3/Bone-Only: No/Brain-Only: No/Visceral: Yes	125 (6,7%)			1000 (100%)		IVc
S2/ER+/HER2-/Grade 1-2/Ductal/T4/Bone-Only: No/Brain-Only: No/Visceral: No	7 (0,4%)		194 (19,4%)	592 (59,2%)	214 (21,4%)	IVc
S2/ER+/HER2-/Grade 1-2/Ductal/T4/Bone-Only: No/Brain-Only: No/Visceral: Yes	57 (3,0%)			1000 (100%)		IVc
S2/ER+/HER2-/Grade 1-2/Lobular/T0-3/Bone-Only: No/Brain-Only: No/Visceral: No	28 (1,5%)			1000 (100%)		IVc
S2/ER+/HER2-/Grade 1-2/Lobular/T0-3/Bone-Only: No/Brain-Only: No/Visceral: Yes	23 (1,2%)			1000 (100%)		IVc
S2/ER+/HER2-/Grade 1-2/Lobular/T4/Bone-Only: No/Brain-Only: No/Visceral: No	4 (0,2%)		30 (3%)	756 (75,6%)	214 (21,4%)	IVc
S2/ER+/HER2-/Grade 1-2/Lobular/T4/Bone-Only: No/Brain-Only: No/Visceral: Yes	6 (0,3%)		48 (4,8%)	952 (95,2%)		IVc
S2/ER+/HER2-/Grade 1-2/Other/T0-3/Bone-Only: No/Brain-Only: No/Visceral: No	5 (0,3%)		710 (71%)	290 (29%)		IVc
S2/ER+/HER2-/Grade 1-2/Other/T0-3/Bone-Only: No/Brain-Only: No/Visceral: Yes	17 (0,9%)		731 (73,1%)	269 (26,9%)		IVc
S2/ER+/HER2-/Grade 1-2/Other/T4/Bone-Only: No/Brain-Only: No/Visceral: Yes	9 (0,5%)		13 (1,3%)	743 (74,3%)	244 (24,4%)	IVc
S2/ER+/HER2-/Grade 3/Ductal/T0-3/Bone-Only: No/Brain-Only: No/Visceral: No	1 (0,1%)		376 (37,6%)	616 (61,6%)	8 (0,8%)	IVc
S2/ER+/HER2-/Grade 3/Ductal/T0-3/Bone-Only: No/Brain-Only: No/Visceral: Yes	40 (2,2%)			999 (99,9%)	1 (0,1%)	IVc
S2/ER+/HER2-/Grade 3/Ductal/T4/Bone-Only: No/Brain-Only: No/Visceral: Yes	17 (0,9%)			987 (98,7%)	13 (1,3%)	IVc
S2/ER+/HER2-/Grade 3/Lobular/T0-3/Bone-Only: No/Brain-Only: No/Visceral: No	2 (0,1%)		41 (4,1%)	952 (95,2%)	7 (0,7%)	IVc

© 2024 Berg T et al. *JAMA Network Open.*

Characteristic Profile	Patients (n=1859)	IVa	IVb	IVc	IVd	Stage from orginal RPA
S2/ER+/HER2-/Grade 3/Lobular/T0-3/Bone-Only: No/Brain-Only: No/Visceral: Yes	1 (0,1%)		15 (1,5%)	985 (98,5%)		IVc
S2/ER+/HER2-/Grade 3/Other/T0-3/Bone-Only: No/Brain-Only: No/Visceral: Yes	1 (0,1%)		205 (20,5%)	794 (79,4%)	1 (0,1%)	IVc
S2/ER+/HER2+/Grade 1-2/Ductal/T0-3/Bone-Only: No/Brain-Only: No/Visceral: No	6 (0,3%)	2 (0,2%)	343 (34,3%)	653 (65,3%)	2 (0,2%)	IVc
S2/ER+/HER2+/Grade 1-2/Ductal/T0-3/Bone-Only: No/Brain-Only: No/Visceral: Yes	26 (1,4%)		52 (5,2%)	948 (94,8%)	0 (0%)	IVc
S2/ER+/HER2+/Grade 1-2/Ductal/T4/Bone-Only: No/Brain-Only: No/Visceral: No	1 (0,1%)		31 (3,1%)	755 (75,5%)	214 (21,4%)	IVc
S2/ER+/HER2+/Grade 1-2/Ductal/T4/Bone-Only: No/Brain-Only: No/Visceral: Yes	10 (0,5%)			1000 (100%)		IVc
S2/ER+/HER2+/Grade 1-2/Lobular/T0-3/Bone-Only: No/Brain-Only: No/Visceral: Yes	2 (0,1%)		41 (4,1%)	957 (95,7%)	2 (0,2%)	IVc
S2/ER+/HER2+/Grade 1-2/Other/T0-3/Bone-Only: No/Brain-Only: No/Visceral: No	2 (0,1%)	3 (0,3%)	340 (34%)	657 (65,7%)		IVc
S2/ER+/HER2+/Grade 1-2/Other/T0-3/Bone-Only: No/Brain-Only: No/Visceral: Yes	2 (0,1%)		177 (17,7%)	821 (82,1%)	2 (0,2%)	IVc
S2/ER+/HER2+/Grade 1-2/Other/T4/Bone-Only: No/Brain-Only: No/Visceral: Yes	1 (0,1%)		19 (1,9%)	874 (87,4%)	107 (10,7%)	IVc
S2/ER+/HER2+/Grade 3/Ductal/T0-3/Bone-Only: No/Brain-Only: No/Visceral: No	1 (0,1%)	2 (0,2%)	810 (81%)	179 (17,9%)	9 (0,9%)	IVc
S2/ER+/HER2+/Grade 3/Ductal/T0-3/Bone-Only: No/Brain-Only: No/Visceral: Yes	11 (0,6%)	194 (19,4%)	719 (71,9%)	87 (8,7%)		IVc
S2/ER+/HER2+/Grade 3/Ductal/T4/Bone-Only: No/Brain-Only: No/Visceral: Yes	4 (0,2%)	49 (4,9%)	377 (37,7%)	574 (57,4%)		IVc
S2/ER+/HER2+/Grade 3/Lobular/T0-3/Bone-Only: No/Brain-Only: No/Visceral: Yes	1 (0,1%)	107 (10,7%)	542 (54,2%)	351 (35,1%)		IVc
S2/ER+/HER2+/Grade 3/Lobular/T4/Bone-Only: No/Brain-Only: No/Visceral: Yes	1 (0,1%)	49 (4,9%)	359 (35,9%)	592 (59,2%)		IVc
S3-4/ER-/HER2-/Grade 1-2/Ductal/T0-3/Bone-Only: No/Brain-Only: No/Visceral: Yes	8 (0,4%)				1000 (100%)	IVd
S3-4/ER-/HER2-/Grade 1-2/Ductal/T4/Bone-Only: No/Brain-Only: No/Visceral: No	1 (0,1%)			1 (0,1%)	999 (99,9%)	IVd
S3-4/ER-/HER2-/Grade 1-2/Ductal/T4/Bone-Only: No/Brain-Only: No/Visceral: Yes	3 (0,2%)			1 (0,1%)	999 (99,9%)	IVd
S3-4/ER-/HER2-/Grade 1-2/Other/T0-3/Bone-Only: No/Brain-Only: No/Visceral: Yes	1 (0,1%)			1 (0,1%)	999 (99,9%)	IVd
S3-4/ER-/HER2-/Grade 1-2/Other/T4/Bone-Only: No/Brain-Only: No/Visceral: Yes	1 (0,1%)			2 (0,2%)	998 (99,8%)	IVd
S3-4/ER-/HER2-/Grade 3/Ductal/T0-3/Bone-Only: No/Brain-Only: No/Visceral: Yes	13 (0,7%)			1 (0,1%)	999 (99,9%)	IVd
S3-4/ER-/HER2-/Grade 3/Ductal/T4/Bone-Only: No/Brain-Only: No/Visceral: Yes	3 (0,2%)				1000 (100%)	IVd
S3-4/ER-/HER2-/Grade 3/Other/T4/Bone-Only: No/Brain-Only: No/Visceral: Yes	2 (0,1%)			1 (0,1%)	999 (99,9%)	IVd
S3-4/ER-/HER2+/Grade 1-2/Ductal/T0-3/Bone-Only: No/Brain-Only: No/Visceral: Yes	7 (0,4%)		1 (0,1%)	580 (58%)	419 (41,9%)	IVd
S3-4/ER-/HER2+/Grade 1-2/Ductal/T4/Bone-Only: No/Brain-Only: No/Visceral: Yes	4 (0,2%)		1 (0,1%)	662 (66,2%)	337 (33,7%)	IVd
S3-4/ER-/HER2+/Grade 1-2/Other/T0-3/Bone-Only: No/Brain-Only: No/Visceral: Yes	1 (0,1%)			594 (59,4%)	406 (40,6%)	IVd

© 2024 Berg T et al. *JAMA Network Open.*

**Characteristic Profile**	**Patients (n=1859)**	**IVa**	**IVb**	**IVc**	**IVd**	**Stage from orginal RPA**
S3-4/ER-/HER2+/Grade 3/Ductal/T0-3/Bone-Only: No/Brain-Only: No/Visceral: Yes	2 (0,1%)		352 (35,2%)	615 (61,5%)	33 (3,3%)	IVd
S3-4/ER-/HER2+/Grade 3/Ductal/T4/Bone-Only: No/Brain-Only: No/Visceral: Yes	2 (0,1%)		205 (20,5%)	751 (75,1%)	44 (4,4%)	IVd
S3-4/ER+/HER2-/Grade 1-2/Ductal/T0-3/Bone-Only: No/Brain-Only: No/Visceral: No	1 (0,1%)		36 (3,6%)	964 (96,4%)		IVc
S3-4/ER+/HER2-/Grade 1-2/Ductal/T0-3/Bone-Only: No/Brain-Only: No/Visceral: Yes	49 (2,6%)			1000 (100%)		IVc
S3-4/ER+/HER2-/Grade 1-2/Ductal/T4/Bone-Only: No/Brain-Only: No/Visceral: Yes	28 (1,5%)			1000 (100%)		IVc
S3-4/ER+/HER2-/Grade 1-2/Lobular/T0-3/Bone-Only: No/Brain-Only: No/Visceral: No	1 (0,1%)			1000 (100%)		IVc
S3-4/ER+/HER2-/Grade 1-2/Lobular/T0-3/Bone-Only: No/Brain-Only: No/Visceral: Yes	14 (0,8%)			1000 (100%)		IVc
S3-4/ER+/HER2-/Grade 1-2/Lobular/T4/Bone-Only: No/Brain-Only: No/Visceral: Yes	7 (0,4%)		341 (34,1%)	659 (65,9%)		IVc
S3-4/ER+/HER2-/Grade 1-2/Other/T0-3/Bone-Only: No/Brain-Only: No/Visceral: Yes	8 (0,4%)			995 (99,5%)	5 (0,5%)	IVc
S3-4/ER+/HER2-/Grade 1-2/Other/T4/Bone-Only: No/Brain-Only: No/Visceral: Yes	1 (0,1%)		296 (29,6%)	704 (70,4%)		IVc
S3-4/ER+/HER2-/Grade 3/Ductal/T0-3/Bone-Only: No/Brain-Only: No/Visceral: Yes	7 (0,4%)		530 (53%)	469 (46,9%)	1 (0,1%)	IVc
S3-4/ER+/HER2-/Grade 3/Ductal/T4/Bone-Only: No/Brain-Only: No/Visceral: Yes	12 (0,6%)			354 (35,4%)	646 (64,6%)	IVc
S3-4/ER+/HER2+/Grade 1-2/Ductal/T0-3/Bone-Only: No/Brain-Only: No/Visceral: Yes	12 (0,6%)			154 (15,4%)	846 (84,6%)	IVc
S3-4/ER+/HER2+/Grade 1-2/Ductal/T4/Bone-Only: No/Brain-Only: No/Visceral: Yes	6 (0,3%)			395 (39,5%)	605 (60,5%)	IVc
S3-4/ER+/HER2+/Grade 1-2/Lobular/T0-3/Bone-Only: No/Brain-Only: No/Visceral: Yes	1 (0,1%)			258 (25,8%)	742 (74,2%)	IVc
S3-4/ER+/HER2+/Grade 1-2/Other/T0-3/Bone-Only: No/Brain-Only: No/Visceral: Yes	1 (0,1%)			269 (26,9%)	731 (73,1%)	IVc
S3-4/ER+/HER2+/Grade 1-2/Other/T4/Bone-Only: No/Brain-Only: No/Visceral: Yes	3 (0,2%)		229 (22,9%)	263 (26,3%)	508 (50,8%)	IVc
S3-4/ER+/HER2+/Grade 3/Ductal/T0-3/Bone-Only: No/Brain-Only: No/Visceral: Yes	12 (0,6%)		622 (62,2%)	378 (37,8%)		IVc
S3-4/ER+/HER2+/Grade 3/Ductal/T4/Bone-Only: No/Brain-Only: No/Visceral: Yes	3 (0,2%)		17 (1,7%)	409 (40,9%)	574 (57,4%)	IVc

© 2024 Berg T et al. *JAMA Network Open.*
