# Supplementary material for: Development and Methodological Validation of a Modified Staging System for de Novo Metastatic Breast Cancer
Source: JAMA Netw Open. 2024 Mar 13;7(3):e242174. doi: 10.1001/jamanetworkopen.2024.2174 (PMC10938173; doi:10.1001/jamanetworkopen.2024.2174)
Supplement: Supplement 2. — Data Sharing Statement [file jamanetwopen-e242174-s002.pdf]

Data Sharing Statement

Berg. Development and Methodological Validation of a Modified Staging System for de Novo Metastatic Breast Cancer. *JAMA Netw Open*. Published March 13, 2024. doi:10.1001/jamanetworkopen.2024.2174

Data
Data available: No

Additional Information
Explanation for why data not available: All data are stored in the DBCG database. The data that generated figures, tables and supplementary data in the published article are not publicly available due to institutional restrictions. The dataset can be made available to qualified researchers through application to the Danish Breast Cancer Group. Please contact dbcg.rigshospitalet@regionh.dk
